# Sexual Dimorphism and Inter-Century Variability in Sella Turcica and Sphenoid Sinus Pneumatization: A Computed Tomography Study of the Romanian Population

**DOI:** 10.3390/diagnostics16182983

**Published:** 2026-09-15

**Authors:** Andra-Ioana Baloiu, Octavian Munteanu, Andrei Dorian Soficaru, Iuliana-Raluca Gheorghe, Andreea-Nicoleta Marinescu, Diana-Alexandra Telembeci, Ioan-Andrei Petrescu, Ștefan Oprea, Florin Mihail Filipoiu

**Affiliations:** 1 Doctoral School, “Carol Davila” University of Medicine and Pharmacy, 020021 Bucharest, Romania; andra-ioana.baloiu@drd.umfcd.ro; 2 Department of Anatomy, “Carol Davila” University of Medicine and Pharmacy, 050474 Bucharest, Romania; 3 University Emergency Hospital of Bucharest, 050098 Bucharest, Romania; 4 “Francisc I. Rainer” Institute of Anthropology, Romanian Academy, 010711 Bucharest, Romania; 5 Department of Marketing and Medical Technology, Faculty of Medicine, “Carol Davila” University of Medicine and Pharmacy, 050474 Bucharest, Romania; 6 Department of Radiology, “Carol Davila” University of Medicine and Pharmacy, 050474 Bucharest, Romania

**Keywords:** dry skull, CT examination, forensic anthropology, sphenoid bone, sella turcica

## Abstract

**Background/Objectives**: Identification of unknown individuals is an essential part of Forensic Medicine. Recent anthropological research has demonstrated subtle but consistent sexual dimorphism in the sella turcica, while indicating comparatively limited population-specific differences in its size, shape, and contour. The aim of this paper is to analyze the variability and sexual dimorphism of the sphenoid bone and to determine if there are statistically significant correlations between the sella turcica dimensions and the degree of pneumatization of the sphenoid sinus. **Materials and Methods**: We analyzed five hundred random unenhanced CT examinations of human dry skulls from the “Francisc I. Rainer” Craniological Collection of the Human Anthropological Institute in Bucharest, Romania. The “Francisc I. Rainer” Craniological Collection is a modern osteological collection of almost 6800 skulls that have been collected and prepared by Prof. Rainer during the first half of the XXth century. Most of the specimens are of Romanian origin, prepared during the interwar period, and over 3000 adult skulls are of known sex and age. Consequently, 600 CT examinations of Romanian patients underwent cerebral CT in the University Emergency Hospital of Bucharest. The overall sample (dried skulls and the skulls of the contemporary living individuals) consisted of 1100 CT examinations of skulls, 619 males and 481 females, with ages between 19 and 93 years. **Results**: we identified statistically significant differences regarding the degree of pneumatization of the sphenoid sinus (SS) between the two groups as a whole, with the conchal and presellar types being more frequent in the dry skull group, and the sellar and postsellar types being more frequent in the patient group. When analyzing whether there were correlations between the degree of pneumatization of the SS and the diameters of the sella turcica (ST), we identified a weak, but statistically significant negative correlation between the sella turcica height (STH) and the postsellar pneumatization type. This suggests that the smaller sellar heights tend to be associated with the postsellar type of SS. Type III interclinoid osseous bridges were more frequent in the contemporary living patients’ group. **Conclusions**: Knowledge of such variability of the sphenoid bone holds great importance in Forensic Sciences, as it may aid in the process of identification of individuals or human remains.

## 1. Introduction

Identification of unknown individuals is an essential part of Forensic Medicine, for both legal and humanitarian reasons [1]. In forensic settings, sex determination depends on the amount and condition of skeletal remains, namely, a greater number of bones improves the likelihood of obtaining reliable sex assessments [2]. In the past years, Multi-Slice Computed Tomography (MSCT) gained an important role in reconstructive identification, i.e., determining a biological profile, whose most important features are sex, stature, and age at the time of death [3]. Radiologically, the skull is among the most valuable skeletal elements for reliable morphometric assessment of sex. Imaging-based approaches—including X-ray, computed tomography (CT), and magnetic resonance imaging (MRI)—have been shown to produce measurements that are consistent with those obtained using calipers, when estimating dry skull dimensions [4].

From a forensic perspective, the basicranium is relatively resilient and, compared with other craniofacial bones, is more often preserved after catastrophic injuries, or through burial and taphonomic processes, because of its position at the skull base, its thickness, and the substantial soft-tissue coverage [5]. Consequently, it may serve as a valuable tool for sex differentiation that can be applied prior to any destructive testing procedures, such as DNA analysis, and may also be useful when genetic testing is not feasible due to degradation—for example, in cases involving extensive heat-induced damage [2,5].

The sphenoid bone is located centrally in the human basicranium, consisting of a three-dimensional body, two lesser wings extending anterolaterally, two greater wings projecting laterally and two anteroinferior pterygoid processes. The body contains the sphenoid sinus (SS); above it lies the sella turcica (ST), a saddle-shaped depression also termed the hypophyseal (or pituitary) fossa, bounded posteriorly by the dorsum sellae (DS) and, anteriorly, by the tuberculum sellae (TS) [6,7]. Three bony projections arise laterally on either side of the sphenoid body, as follows: at the medial end of the lesser wing of the sphenoid, the anterior clinoid process provides attachment for the free margin of the tentorium cerebelli; the middle clinoid processes arise lateral to the TS, whereas the posterior clinoid processes project superiorly and laterally from the DS [8]. Bony osseous bridges connecting these processes form additional foramina in the middle cranial fossa, on either side of the sphenoid body, interacting with neurovascular structures, such as the internal carotid artery (ICA) and the nerves passing through the cavernous sinus [9].

Recent anthropological research has demonstrated subtle but consistent sexual dimorphism in the sella turcica—males typically show larger, deeper sellar depressions—while indicating comparatively limited population-specific differences in its size, shape, and contour across African, Asian, and European groups—reflecting possible environmental adaptation and/or neutral genetic drift, thereby supporting its utility for sex estimation [10]. However, there is an abundance of the literature regarding the morphological and morphometric characteristics of the sella turcica, both using radiographic [11,12,13] or computed tomography methods [14,15,16,17]. One study revealed that visually comparing sphenoid sinus anatomy on computed tomography scans can assist personal identification when antemortem dental records, DNA, or fingerprints are unavailable [18].

Moreover, accurate knowledge about sella turcica and sphenoid sinus anatomy, as well as its variability, is essential for preoperative planning and intraoperative navigation, because it directly affects surgical access when using the endoscopic endonasal transsphenoidal approach (EETA) [19]. Sellar bridges are clinically important because of their close proximity to the ICA and cavernous sinus, where they may cause neurovascular compression or traction, and complicate anterior or middle clinoidectomy, increasing the risk of carotid injury during skull base surgery [9].

Nevertheless, the existing studies are limited by small sample sizes, heterogeneous parameter selection and coverage of individual population groups. To the best of our knowledge, despite the fact that several studies have evaluated sexually dimorphic features of the sphenoid, most existing studies were based on relatively small sample sizes of either patients or skulls, and only a limited number performed comparative analyses between two populations from different centuries. Moreover, this is the first study to assess sexual dimorphism of the sphenoid bone in the Romanian population, based on specimens from the “Francisc I. Rainer” Craniological Collection as well as contemporary patients.

The “Francisc I. Rainer” Craniological Collection is a modern osteological collection of almost 6800 skulls that have been collected and prepared by Prof. Rainer during the first half of the XXth century. Most of the specimens are of Romanian origin, prepared during the interwar period, and over 3000 adult skulls are of known sex, age, cause and date of death, as they were prepared by Prof. Rainer himself from cadavers from hospitals and morgues.

In this study, we analyzed the degree of pneumatization of the sphenoid sinus, the antero-posterior and cranio-caudal diameters of the sella turcica, and the presence of interclinoid bridges on CT scans of crania from the Craniological Collection, and on CT scans of contemporary patients who underwent cerebral CT for various medical reasons. The aim of this study is to analyze the variability and sexual dimorphism of the sphenoid bone by (1) determining the variability of the pneumatization of the sphenoid sinus and interclinoid osseous bridges and determining the dimensions of sella turcica, comparing their variation with sex and age across both samples of CT examinations from the Romanian population, (2) assessing whether there are statistically significant differences between the two samples of populations almost 100 years apart, and (3) determining if there are statistically significant correlations between the sella turcica dimensions and the degree of pneumatization of the sphenoid sinus.

## 2. Materials and Methods

### 2.1. Specimen Selection

This study was designed as a retrospective cross-sectional observational morphometric study based on the analysis of cranial computed tomography examinations.

Five hundred randomly selected unenhanced CT scans of dry human skulls from the “Francisc I. Rainer” Craniological Collection of the Human Anthropological Institute in Bucharest, Romania, were examined. Randomization was performed, by applying a random number generator to a digitized inventory list, from which the selected subset of specimens was retrieved. Each skull in the sample had prior anthropological identification: 216 were recorded as female and 284 as male, with ages at death ranging from 20 to 82 years. All specimens are of Romanian origin and date from the interwar period (the crania included in this study were prepared between 1921 and 1943). This retrospective analysis was conducted in accordance with the Declaration of Helsinki. The Ethics Committee of the “Carol Davila” University of Medicine and Pharmacy in Bucharest (approval no. 14355; 30 May 2024), the Ethics Committee of the “Francisc I. Rainer” Institute of Anthropology (approval no. 847; 29 May 2024) and the Ethics Committee of the University Emergency Hospital of Bucharest (approval no. 32986; 27 May 2024) approved the digitalization of the Craniological Collection.

Consequently, 600 CT examinations of Romanian patients who underwent cerebral CT in the University Emergency Hospital of Bucharest between 1 March 2023 and 1 March 2024 were examined. The first 500 patients were selected on condition that they had as much as possible the same sex and same age to each of the selected crania from the Collection. The last 100 patients were equally distributed regarding sex (50 males and 50 females) and age (10 males and 10 females for each age: 19–20 years, 30 years, 40 years, 50 years, 60 years). The final sample included 265 females and 335 males, aged between 19 and 93 years. Exclusion criteria were: technically suboptimal scans, motion or metallic artifacts, severe traumatic or destructive lesions that impaired the evaluation of the sphenoid bone, and congenital malformations of the skull base.

The overall sample (dried skulls and the skulls of the contemporary living individuals) consisted of 1100 CT examinations of skulls, 619 males and 481 females, with ages between 19 and 93 years.

### 2.2. Imaging

All CT scans of the human dry skulls were acquired on a Canon Aquilion One 64-slice multi-detector computed tomography (MDCT) scanner (Canon Medical Systems, Otawara, Tochigi, Japan) at the Department of Radiology, University Emergency Hospital of Bucharest. Multiple acquisitions with varying parameters were performed to determine the optimal settings for image quality and anatomical detail. The final protocol consisted of 120 kVp tube voltage and 19 mA tube current. It was selected and confirmed by three investigators to provide appropriate contrast and signal-to-noise ratio. After establishing imaging parameters, additional CT acquisitions were conducted, in order to identify the best support method for holding skulls in anatomical position without degrading 3D reconstructions. We evaluated cardboard boxes, various CT-compatible foam head supports, radiolucent positioning sponges, and polystyrene foam rings. Ultimately, skulls were supported by resting the occipital bone on a polystyrene foam circular ring (15 cm as internal diameter), chosen for its very low density (≈970 Hounsfield units), compared with surrounding air (≈990 to −1000 HU). Routine air calibrations were performed at manufacturer-recommended intervals to preserve HU accuracy and correct for potential detector drift. Skulls were positioned using laser alignment: the axial laser along the superior orbital margins, the sagittal laser on the internasal and intermaxillary sutures and the midline of the foramen magnum, and the coronal laser through both external acoustic meatus. Multiplanar 2D reconstructions were generated in axial, coronal, and sagittal planes using a bone window with 0.5 mm as slice thickness. Additionally, 3D reconstructions were produced to improve assessment of the skull base. All scans were reviewed by three investigators (A.I.B., A.N.M. and D.A.T.) on dedicated workstations.

All CT scans of the patients were acquired at the Department of Radiology and the Compartment of Emergency Radiology within the University Emergency Hospital of Bucharest, on several CT scanners, including a Canon Aquilion One 64-slice MDCT scanner (Canon Medical Systems, Otawara, Tochigi, Japan) and a Philips Incisive 128-slice MDCT scanner (Philips Healthcare, Best, The Netherlands). The raw data acquisition parameters included a fixed tube voltage of 120 kV, while the tube current (mA) was dynamically regulated using automatic exposure control, with 1 mm for collimation. CT image reconstruction was performed with a slice thickness of 1 mm and increment of 1 mm, using bone kernels.

### 2.3. Sphenoid Sinus Evaluation

In 5 of the dry skulls, the body of the sphenoid bone was absent or damaged, so they were excluded from the analysis.

**Figure 1:**
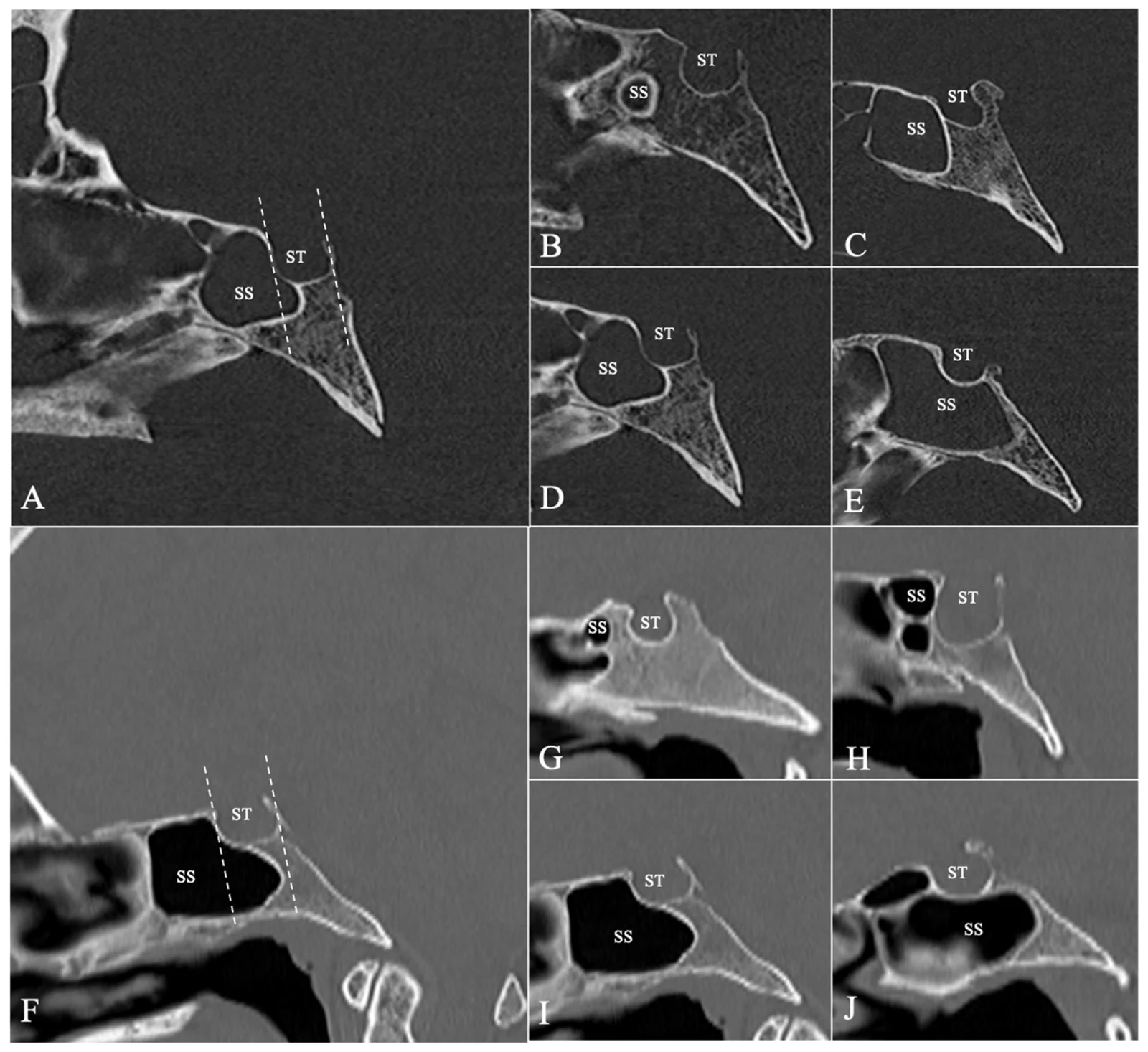
Median sagittal sections through the crania from the “Francisc I. Rainer” Craniological Collection (**A**–**E**) and contemporary living patients (**F**–**J**) depicting the degree of sagittal pneumatization of the sphenoid sinus (SS) in relation to the anterior and posterior walls of the sella turcica (ST) (**A**,**F**). (**B**,**G**)—Chonchal type, in which the SS is separated from the ST by a thick, bony wall; (**C**,**H**)—presellar type: the SS extends posteriorly to the anterior wall of the ST; (**D**,**I**)—sellar type: the SS extends posteriorly between the ST’s anterior and posterior walls; (**E**,**J**)—postsellar type: the SS extends posteriorly beyond the posterior wall of the ST. The dotted lines indicate the anterior (left) and posterior (right) walls of the ST.

Based on the classification proposed by Güldner [20], we evaluated the anterio-posterior extension of the SS pneumatization in the sagittal plane, as follows (Figure 1):

- Chonchal type: The sphenoid sinus is characterized by a very small sinus, separated from the sella turcica by a thick, hard bony wall;
- Presellar type: The sphenoid sinus extends posteriorly to the anterior border of the sella, but not invading the sellar floor;
- Sellar type: The sphenoid sinus extends posteriorly between the sella’s anterior and posterior walls;
- Postsellar type: The sphenoid sinus extends posteriorly beyond the posterior wall of the sella, with possible extensions inside the dorsum sellae or the clivus.

When encountering different pneumatization degrees between the left and right sphenoid sinuses, the greater extension was taken into account.

### 2.4. Sella Turcica Evaluation

Because of the dry skull preparation method, 259 specimens presented an absent/damaged dorsum sellae or body of the sphenoid sinus, making the morphometric analysis of the sella turcica very difficult. Thus, the final sample of dry crania for performing the measurements included 241 skulls. There were 105 females (43.5%) and 136 males (56.5%), with a mean age of 52.37, SD = 13.09. The overall sample included 841 CT examinations, with 370 (44%) females and 471 (56%) males.

**Figure 2:**
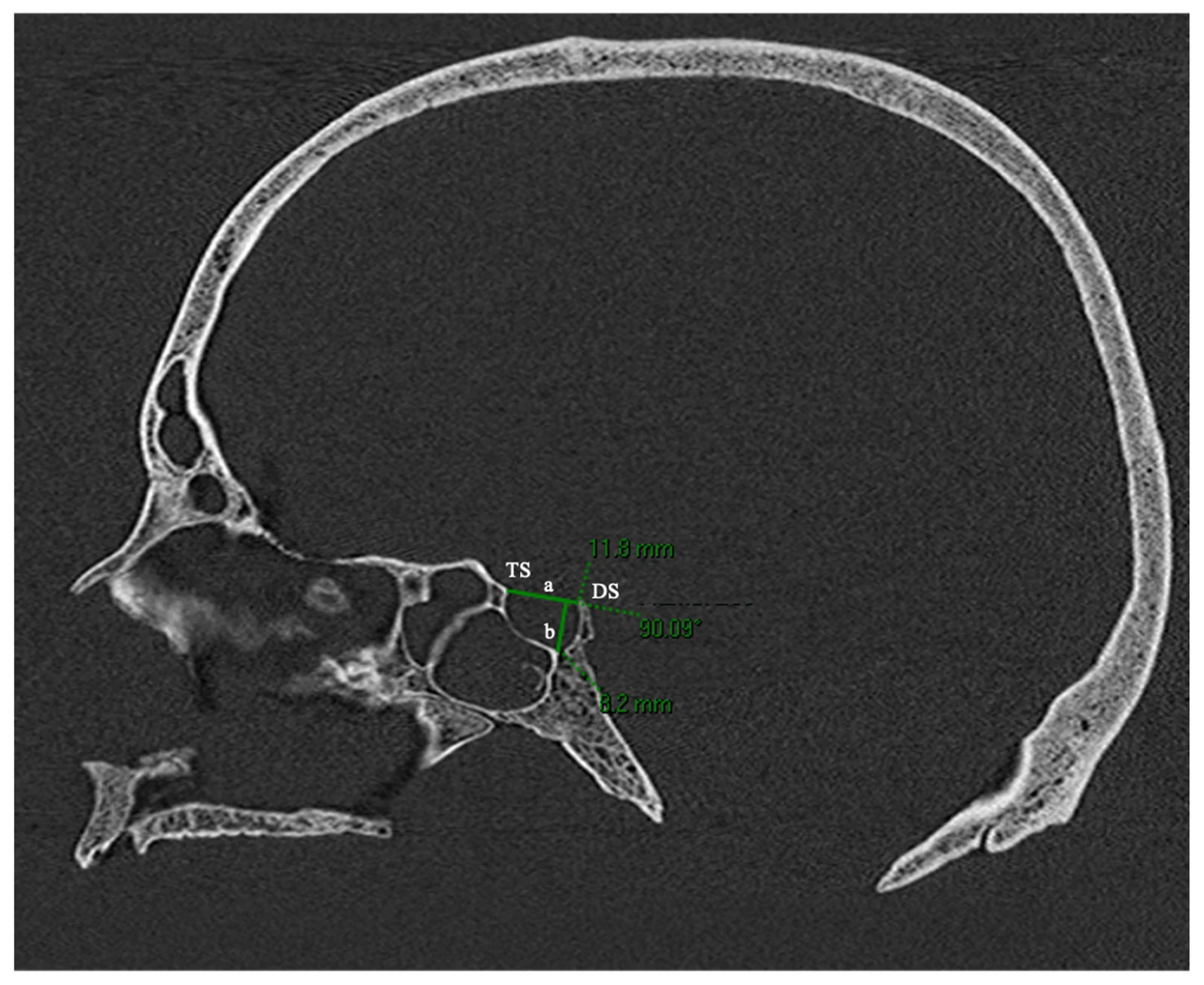
Median sagittal sections through a cranium from the “Francisc I. Rainer” Craniological Collection. TS—Tuberculum sellae; DS—dorsum sellae; a—distance between the TS and DS, considered the anterio-posterior (AP) diameter of the sella turcica; b—the perpendicular line from the TS–DS line (a) to the deepest point of the ST floor, accounting for the height of the ST.

Although there is a consistent body of the literature that focuses on the analysis of the dimensions of the sella turcica [10,11,12,13,14,15,16,17], there is an increased variability between the parameters that are measured. In this study, measurements were performed on the MPR reconstructions of the mediosagittal plane. In both samples, the mediosagittal plane was manually adjusted and confirmed in all three orthogonal planes, as the plane that passes sagitally through the nasal septum and crista galli, the interhemispheric fissure and falx cerebri (when present), and through the middle of the clivus and foramen magnum. Two landmarks were carefully placed: the tuberculum sellae (TS) and the dorsum sellae (DS). The distance between the two landmarks was considered the anterio-posterior (AP) diameter of the sella turcica. The perpendicular line from the TS–DS line to the deepest point of the ST floor was measured, accounting for the height of the sella turcica (STH) (Figure 2).

### 2.5. Interclinoid Osseous Bridges Evaluation

**Figure 3:**
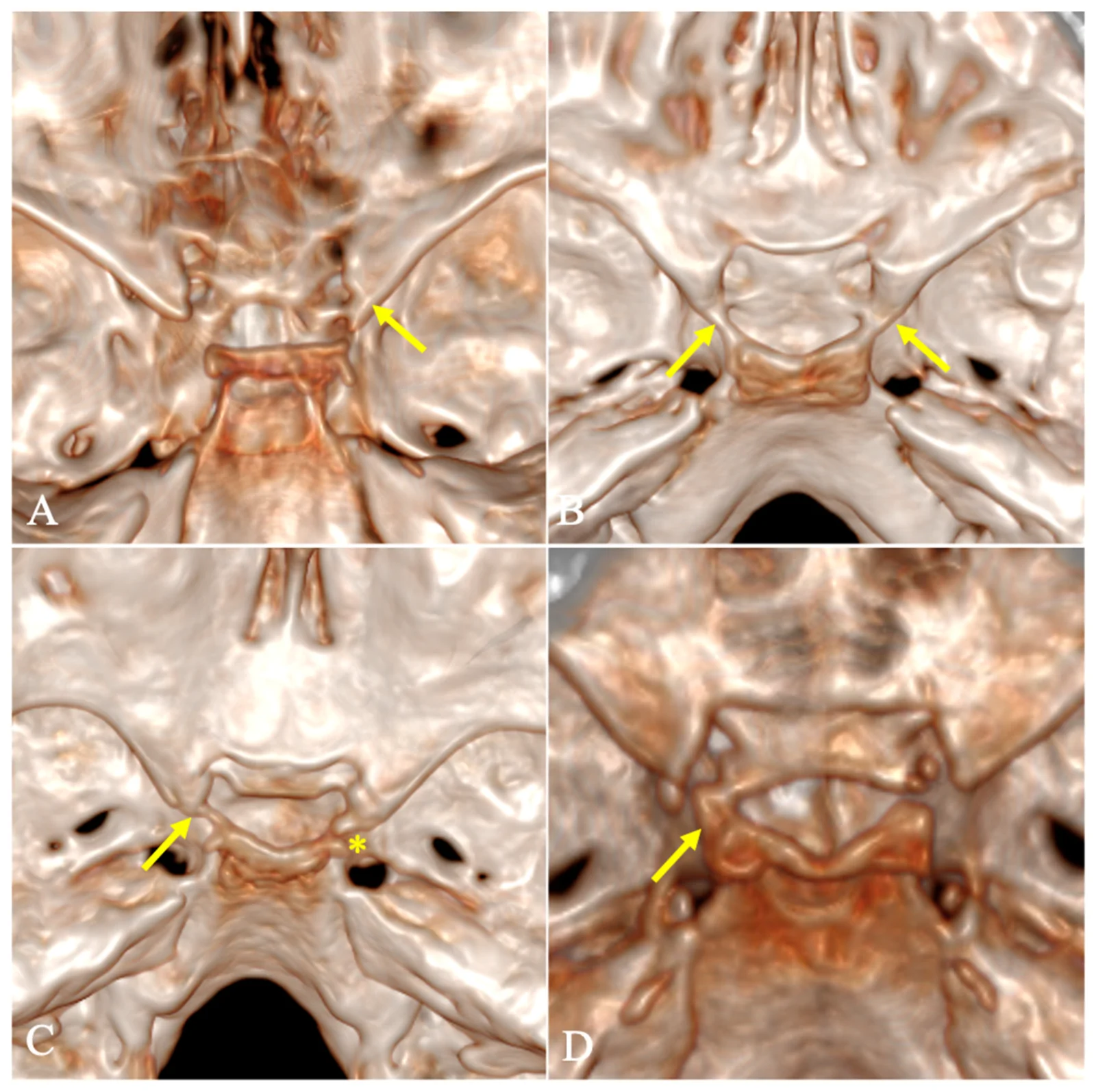
Three-dimensional reconstructions of crania from the “Francisc I. Rainer” Craniological Collection (**B**,**C**) and contemporary living patients (**A**,**D**), depicting the internal surface of the skull base, after digitally removing the calvaria. The arrows represent: (**A**)—right-sided Type 1 complete osseous bridge between the anterior and middle clinoid processes; (**B**)—bilateral Type II complete osseous bridge between the anterior, middle, and posterior clinoid processes; (**C**)—left-sided Type III complete osseous bridge between the anterior and posterior clinoid processes; and (**D**)—left-sided Type IV complete osseous bridge between the middle and posterior clinoid processes. Note the right-sided Type III contact osseous bridge between the anterior and posterior clinoid processes (*).

The presence of interclinoid bridges was evaluated on both bidimensional MPR reconstructions in the three anatomical planes and 3D reconstructions of the skull, after digitally removing the calvaria. We classified the presence of the osseous bridges according to the classification proposed by Archana et al. [21] (Figure 3):

- Type I: Osseous bridge between the anterior and middle clinoid processes (carotid-clinoid foramen);
- Type II: Osseous bridge between the anterior, middle, and posterior clinoid processes (complete sellar bridge);
- Type III: Osseous bridge between the anterior and posterior clinoid processes (interclinoid bridge or canal of Gruber) [22];
- Type IV: Osseous bridge between the middle and posterior clinoid processes.

Both complete osseous bridges were taken into consideration, i.e., a complete fusion between two bony bars and contact osseous bridges, in which a millimetric-dividing line or suture was present between the bony bars.

### 2.6. Examiner Consistency

One hundred and fifty randomly selected crania were observed again by the same investigators one month after the first round of observations. Examiner consistency was assessed for all variables. Variables were highly similar between the first and second rounds of observations, with interclass correlation coefficients (ICC) of 0.84–0.97.

### 2.7. Statistical Analysis

The collected data was processed and analyzed with SPSS (Statistical Package for the Social Sciences ver.20; IBM Corporation, Armonk, NY, USA). For the description of the qualitative variables, the frequencies and percentages were used, while the quantitative data was presented by using the mean and the standard deviation. The Shapiro–Wilk test was used to check the normality distribution of the quantitative variables (*p* < 0.05). In the case of identifying differences between quantitative variables by a qualitative variable and the quantitative variables also having nonparametric distributions, the Mann–Whitney U test was employed, whereas in order to identify differences between qualitative groups, the Chi-square test was used. In addition, the correlations between a qualitative and a quantitative variable were determined by point-biserial correlation, or the Pearson correlation for assessing the correlation between quantitative variables. We analyzed the relationship between ST dimensions and age using correlation and regression analyses to assess age-related changes. The statistically significant threshold was at *p* < 0.05.

## 3. Results

Five hundred CT examinations of dry skulls (216 females, 284 males) with ages ranging between 20 and 82 years (mean age of 46.73, SD = 15.818, 43.2% females, 56.8% males) were included in the present study. Six hundred CT examinations of contemporary living patients (265 females, 335 males) with ages ranging between 19 and 93 years (mean age of 46.46, SD = 16.306, 44.2% females, 55.8% males) were included in this study. The overall sample (dry skulls and skulls of contemporary living patients) included 1100 included CT examinations of skulls, with the ages between 19 and 93 years, a mean age of 46.59, 43.7% females and 56.3% males. There were no statistically significant differences between the two groups regarding sex distribution (Chi-square = 0.10, *p* = 0.74 > 0.05).

### 3.1. Sphenoid Sinus Evaluation

In the dry skull group, the most frequent pneumatization type of the SS was the sellar type in 41.40% (n = 205) of the crania while the least frequent was the conchal type, in 3.80% (n = 19) of the crania. In the contemporary living patients’ group, the most frequent pneumatization type of the SS was the postsellar type in 46.00% (n = 276) of the crania. The results are summarized in Table 1. There were no statistically significant differences between the degree of pneumatization of the SS and sex or age. However, we identified statistically significant differences regarding the degree of pneumatization of the SS between the two groups as a whole (Chi-square = 19,84, *p* = 0.001), with the conchal and presellar types being more frequent in the dry skull group, and the sellar and postsellar types being more frequent in the patients’ group.

### 3.2. Sella Turcica Evaluation

In the dry crania group, the mean STH was 7.64 mm (SD = 1.27, min = 3.30, max = 11.40), while the mean AP diameter of the ST was 10.32 mm (SD = 1.65, min = 6.40, max = 15.40). In the contemporary living patients’ group, the mean STH was 8.22 mm (SD = 1.33, min = 5.00, max = 16.30), while the mean AP diameter of the ST was 10.00 mm (SD = 1.67, min = 5.40, max = 17.00). Thus, at the overall sample level, the mean STH was 8.05 mm (SD = 1.34, min = 3.30, max = 16.30) and the mean AP diameter of the ST was 10.09 mm (SD = 1.67, min = 5.40, max = 17.00). These results are depicted in Table 2 and Table 3.

The Mann–Whitney U test revealed significant differences between the dry skull and contemporary living patients’ groups for the STH (MW = 55,506.00; *p* = 0.001) and AP diameter of ST (MW = 63,978.50; *p* = 0.009). The dry crania group showed a smaller STH (7.64 vs. 8.21) and a larger AP diameter of ST (10.32 vs. 10.00), compared to the contemporary living patients’ group.

The point-biserial correlation coefficient revealed a weak, but statistically significant negative correlation between the female sex and the AP diameter of ST across all three groups (dry skull group: r = −0.18, *p* = 0.004; MW = 5462.00, *p* = 0.002; contemporary living patients’ group: r = −0.18, *p* = 0.001; MW = 35,149.50, *p* = 0.001; overall sample: r = −0.18, *p* = 0.001; MW = 68,406.00, *p* = 0.001), indicating that females tend to have smaller AP diameters of ST, regardless of the age of the examined group. There were no statistically significant differences regarding the STH and sex.

When analyzing the diameters of ST in relation with age, it was registered a weak, statistically significant negative correlation between the STH and ages under 47 years across all three groups (dry skull group: r = −0.19, *p* = 0.003; MW = 5751.50, *p* = 0.01; contemporary living patients’ group: r = −0.10, *p* = 0.008; MW = 40,047.50, *p* = 0.03; overall sample: r = −0.13, *p* = 0.001; MW = 76,289.50, *p* = 0.002), indicating that greater STH was associated with an age of under 47 years. There were statistically significant differences regarding the AP diameter of ST and age in the dry crania group: the AP diameter of ST increases proportionally with age (r = 0.13, *p* = 0.03). However, no statistically significant differences regarding the AP diameter of ST and age in the contemporary living patients’ group was identified (r = 0.05, *p* = 0.21).

When analyzing whether there were correlations between the degree of pneumatization of the SS and the diameters of the ST, we identified weak, but statistically significant positive correlations between the STH and the first three types of SS pneumatization (i.e., chonchal, presellar, sellar), and a weak, but statistically significant negative correlation between the STH and the postsellar pneumatization type. This suggests that the smaller sellar heights tend to be associated with the postsellar type of SS, prone to hyperpneumatization. There were no statistically significant differences regarding the AP diameter of ST and the SS pneumatization type. These results are presented in Table 4.

### 3.3. Interclinoid Osseous Bridges Evaluation

In the dry crania group, the most frequent osseous bridge was Type I, in 12% (n = 60) of the skulls. In 4.6% (n = 23) of the skulls, this bar was present bilaterally, whereas in 8.6% (n = 41) of the skulls it was unilateral. In the contemporary living patients’ group, the most frequent osseous bridge was Type I, with 15% (n = 90). The prevalence was similar between the latter two types: 9.7% (n = 58) for Type II, and 9.5% (n = 57) for Type III osseous bridges.

Finally, of the 1100 skulls, 13.6% (n = 150) had a Type I interclinoid osseous bridge. In 4.4% (n = 48) of the skulls this bar was present bilaterally, whereas in 9.3% (n = 102) of the skulls it was unilateral. Type II osseous bridges were encountered in 8.3% (n = 91) of the skulls, in 3.9% (n = 43) bilaterally and 4.4% (n = 48) unilaterally. Type III osseous bridges were presented in 5.1% (n = 67) of the skulls, in 1.5% (n = 17) bilaterally and 4.6% (n = 50) unilaterally. These results are summarized in Table 5. The Type IV osseous bridge was the rarest: only 0.6% (n = 7) out of the 1100 analyzed skulls presented it unilaterally, two (0.4%) of them in the dry skull group and five (0.8%) in the contemporary living patients’ group.

When performing the Chi-square test, a statistically significant difference between the two groups regarding the Type III osseous bridge was present: Type III interclinoid osseous bridges were more frequent in the contemporary living patients’ group (Chi-square = 26.82, *p* = 0.001).

## 4. Discussion

### 4.1. Sphenoid Sinus Evaluation

In this study, when analyzing the dry skull group, the conchal type was present in 3.8% (n = 19), presellar type in 15.6% (n = 77), sellar type in 41.4% (n = 205) and postsellar type in 39.2% (n = 194). In contrast, analyzing 48 adult dry crania, Kayalioglu et al. [23] encountered the conchal type in 4.2%, presellar type in 8.3%, sellar type in 60.4% and postsellar type in 27.1% of the specimens. There is a scarcity of research analyzing the sphenoid sinus in dry crania using CT; one study of such matter [24] analyzed 80 CT examinations of dry skulls and encountered the sellar type of pneumatization in 68 skulls (85%), presellar type in eight (10%) and postsellar type in four skulls (5%). In a similar study on 51 dry skulls using cone-beam CT (CBCT), the conchal pneumatization was registered in 2%, presellar in 24%, sellar in 41% and postsellar in 33% of total sinuses [25].

The sellar type of pneumatization was most frequently encountered in all the studies involving dry crania; however, the temporal provenance (age) of the crania was not mentioned.

Regarding the contemporary living patients’ group, 600 cerebral CT examinations were analyzed, and the sellar and postsellar types were more frequently encountered, which was in accordance with previous research. For instance, one study conducted on 300 patients discovered similar prevalence: 0.7% conchal, 6.6% presellar, 30% sellar, and 62.7% postsellar [22], while another large study conducted on an Indian population group reported similar high frequencies of the “incomplete sellar” (equivalent to sellar type) in 22.2% (n = 111), and “complete sellar” (equivalent to postsellar type) in 76.6% (n = 383) [26].

Another small study on a Romanian sample encountered the postsellar type in only 2% (n = 1) of the patients, while the sellar type was the most frequent (82%, n = 41) [27], but these results may be attributed to the small studied sample. However, the studies mention no significant differences in what concerns gender.

### 4.2. Sella Turcica Evaluation

The morphometry of sella turcica has been a subject of interest for many researchers. However, there is great variability in the selected landmarks and the various distances measured between them. In this study, two landmarks were carefully placed on mediosagittal sections: the tuberculum sellae (TS) and the dorsum sellae (DS). The distance between the two landmarks was considered the AP diameter of the ST. The perpendicular line from the TS–DS line to the deepest point of the ST floor was measured, accounting for the STH. In this study, in the dry crania group, the mean STH was 7.64 mm, while the mean AP diameter of the ST was 10.32 mm. There are few studies in the literature analyzing the ST in dry crania using CT. One study analyzed CT examinations of 100 adult dry skulls and determined the mean AP diameter (TS–DS distance) to be 10.31 mm, and the ST height to be 6.33 mm [28], which have similar values to the ones presented in this study.

In the contemporary living patients’ group, the mean STH was 8.22 mm, while the mean AP diameter of the ST was 10.00 mm. Keleş et al. [29] conducted a multiparametric analysis of the ST, in which the mean AP diameter of the ST (“sella length SL”) was 9.65 mm in females and 9.74 mm in males, similar to our mean values in females of the living patients’ group (9.65 mm in females and 10.28 mm in males). Al-Dwairy et al. [30] analyzed CT examinations of 100 patients and determined the length of the ST (i.e., the AP diameter of ST), which ranged from 4 mm to 14.3 mm in men and from 7.2 mm to 13.8 mm in women, with a mean (SD) length of 9.95 (2.24) mm and 9.97 (1.49) mm in men and women, respectively. However, the two previously mentioned studies have not encountered any statistically significant differences between the two sexes regarding this measurement. In contrast, in this study, it was determined that females tend to have smaller AP diameters of ST, with no significant differences regarding the STH, regardless of the age of the examined cohort. This was in accordance with the findings of a meta-analysis, which focused on sella turcica X-ray features for sex estimation in forensic anthropology [12]. Moreover, it was concluded that the AP diameter of ST increases with the increase in age (r = 0.13, *p* = 0.03). Similarly, Lazzeroni et al. [31] concluded that, in females, age correlated positively with ST length and depth (*p* < 0.01).

### 4.3. The Relationship Between the Sphenoid Sinus and Sella Turcica

The relationship between sella morphology and sphenoid sinus pneumatization has not been thoroughly investigated in the literature. Lazzeroni et al. [31] determined that in males, pneumatization of the greater wings was associated with reduced ST antero-posterior diameter and depth (*p* < 0.01); in females, pneumatization of the pterygoid processes correlated with lower ST depth (*p* < 0.01), and in both sexes, SS volume correlated negatively with ST depth (*p* < 0.01). In this study, although a volumetric analysis of the SS was not performed, it was concluded that the larger heights of ST tend to associate with the conchal, presellar and sellar types of SS pneumatization, and that there is a disproportionate relationship between the STH and pneumatization of SS; smaller sellar heights tend to be associated with the postsellar type of SS, being prone to hyperpneumatization (*p* = 0.001).

### 4.4. Interclinoid Osseous Bridges Evaluation

Regarding the interclinoid osseous bridges, Type I bridges were most frequently encountered in 12% (n = 60) of the dry skulls (4.6%, n = 23, bilaterally, and 7.4%, n = 37, unilaterally), and 15% (n = 90) of the contemporary living patients’ group (4.2%, n = 25, bilaterally, and 10.8%, n = 65, unilaterally). Analyzing 315 CT patient examinations, Nikolova et al. [31] encountered the Type 1 bridges (“complete and contact types”, similar to this study) in 9.5% (7.8% in males and 11.1% in females). Similarly, we identified these bridges in 11.1% in females, while in males the percentage was higher (12.7%). The Type I bridges have been a subject of interest to many researchers, with various detected prevalences in different population groups: India [21] with 5.2%, Turkey [32] with 8.8% for the complete and contact types, or Greece with 23.6% [33] for the complete type. A previous investigation of Bulgarian males from the beginning of the 20th century [34] revealed a frequency of 8.2% (nine out of 110 dry skulls) for this type of interclinoid bridges, a lower prevalence compared to our study findings.

Archana et al. [21] encountered the Type II and III bridges (complete and contact types) in only 1.6% (n = 4 skulls) unilaterally each. Similarly, we encountered Type III bridges in 2% (n = 10) of the dry crania, but with a prevalence of 1% bilaterally. Moreover, it was concluded that Type III interclinoid osseous bridges were more frequent in the contemporary living patients’ group, when comparing to the dry crania.

In general, the findings of the present study are concordant with those previously reported in the literature. Nevertheless, the discrepancies observed may reflect several methodological and sampling-related factors. First, investigations based on dry crania remain limited, and the available studies commonly include relatively small samples, regardless of whether the assessments were performed macroscopically or by computed tomography. Second, the present study may be subject to a selection and population bias, as it applied a retrospective design and analyzed CT examinations obtained in a clinical setting, rather than from a population-based sample. Third, interstudy variability may result from differences in methodology, including macroscopic versus CT-based evaluation, as well as variations in scanner types, acquisition parameters, protocols, image quality, and anatomical landmark selection, as even minor differences in measurement and data-collection procedures may influence the reported outcomes. Accordingly, only after these methodological and technical variables have been sufficiently standardized, may these variations be attributed to ancestry, genetic background, environmental factors or secular changes.

The biological implications of these findings should also be interpreted with caution. The observed anatomical variations do not provide clear-cut classifications, but rather represent probabilistic patterns with limited individual-level certainty. Consequently, they should only be regarded as supplementary anatomical characteristics, rather than standalone evidence in forensic anthropological assessments.

From a clinical perspective, however, the findings may contribute to a more comprehensive understanding of skull-base anatomical variability, symptomatology, as well as its potential relevance to surgical planning and intervention. For example, Type I sellar bridges may enclose and compress the ICA, accounting for headaches or other compression symptoms, including muscle disfunction due to III^rd^ cranial nerve compression [33]. Moreover, their presence increases risks and complicates these interventions [35]. A detailed knowledge of each patient’s unique anatomy of pneumatization of the SS also directly affects the outcome of skull base surgery, as the sellar and postsellar types of pneumatization allow for easier access to the posterior fossa than the chonchal and presellar types [36].

Despite the existing body of the literature on sphenoid sinus and sella turcica morphology, this is the first study to systematically characterize the anatomical variability and sexual dimorphism of these structures in the Romanian population. Our investigation aims to address a significant gap in the comparative anthropological literature by providing population-specific data that enables more nuanced understanding of how genetic ancestry, geographic origin and secular changes may contribute to the morphological diversity observed across different ethnic and geographic populations.

### 4.5. Limitations and Future Directions

Given the large number of potential morphological parameters that may be evaluated in this important anatomical region, a key limitation of the present study is that we did not perform a more comprehensive multiparametric assessment. In particular, we were not able to include advanced quantitative approaches such as SS volumetry, which could have provided additional discriminatory information regarding sexual dimorphism as well as aid in surgical management of patients with skull base pathology.

Another limitation refers to the representativeness and breadth of the sample. Although the “Francisc I. Rainer” Craniological Collection contains more than 6800 cranial specimens, only a small fraction was examined in this study.

Extending the number of specimens and incorporating additional quantitative parameters in future work would likely strengthen the robustness of the findings and improve their generalizability across the Romanian population. Moreover, the use of advanced three-dimensional reconstructions such as cinematic volume rendering (cVR) or photo realistic volume rendering (PRVR) techniques may prove useful in identifying such anatomical landmarks. Nonetheless, the technology advancement in AI-based morphometric analysis could enable more efficient data collecting and facilitate comparison of complex anatomical features.

## 5. Conclusions

The principal contribution of this study lies in establishing of baseline morphometric and morphological standards for the sphenoid sinus and sella turcica in the Romanian population, and quantifying the prevalence of these anatomical variants, filling a significant gap in the comparative studies in the field. These findings have direct implications for the Romanian population, by providing population-specific reference values that may aid clinical diagnosis and treatment planning in neurosurgical contexts. While the study documents anatomical variation and sexual dimorphism, its forensic anthropological utility remains limited; however, the established reference standards may assist in population affinity assessment in future forensic casework.

Our results show that the conchal and presellar types of SS pneumatization are statistically more frequent in the dry skull group from the interwar period, while the sellar and postsellar types are statistically more frequent in the contemporary living patients’ group. Women tend to have smaller AP diameters of ST, while there were no statistically significant differences regarding the STH and sex. The AP diameter of ST tends to increase with the increase in age. Larger ST heights tend to associate with the conchal, presellar and sellar types of SS pneumatization, while smaller ST heights tend to associate with the postsellar type of SS, being prone to hyperpneumatization. Type III interclinoid osseous bridges were significantly more frequent in the contemporary living patients’ group.

## Figures and Tables

**Table 1:** Frequency of degrees of pneumatization of the SS, by group and by sex.

		Dry Skull Group			Patient Group			Complete Group		
		Total no. (%)	Female no. (%)	Male no. (%)	Total no. (%)	Female no. (%)	Male no. (%)	Total no. (%)	Female no. (%)	Male no. (%)
**Pneumatization type**	**Conchal**	19 (3.80%)	5 (1.01%)	14 (2.83%)	6 (1.00%)	4 (0.67%)	2 (0.34%)	25 (2.28%)	9 (0.82%)	16 (1.47%)
	**Presellar**	77 (15.60%)	35 (7.06%)	42 (8.48%)	59 (9.83%)	28 (4.66%)	31 (5.16%)	136 (12.43%)	63 (5.75%)	73 (6.67%)
	**Sellar**	205 (41.40%)	91 (18.40%)	114 (23.03%)	259 (43.17%)	124 (20.66%)	135 (22.50%)	464 (42.37%)	215 (19.63%)	249 (22.73%)
	**Postsellar**	194 (39.20%)	82 (16.56%)	112 (22.63%)	276 (46.00%)	109 (18.67%)	167 (27.84%)	470 (42.92%)	191 (17.45%)	279 (25.48%)
**Total**		495 (100%)	213 (43.03%)	282 (56.97%)	600 (100%)	265 (44.16%)	335 (55.84%)	1095 (100%)	478 (43.65%)	617 (56.35%)

**Table 2:** The mean dimensions of the sella turcica height (STH)—comparison by group and sex.

STH	Dry Skull Group			Patient Group			Total		
	Total	Female	Male	Total	Female	Male	Total	Female	Male
**Mean (mm)**	7.64	7.72	7.58	8.22	8.25	8.18	8.05	8.10	8.01
**St. Dev.**	1.27935	1.15204	1.37029	1.33506	1.31880	1.34717	1.34456	1.29417	1.38129
**Min (mm)**	3.30	5.00	3.30	5.00	5.00	5.30	3.30	5.00	3.30
**Max (mm)**	11.40	11.40	11.00	16.30	14.90	16.30	16.30	14.90	16.30
**Total (no.)**	241	105	136	600	265	335	841	370	471

**Table 3:** The mean dimensions of the AP diameter of the ST—comparison by group and sex.

AP Diameter of ST	Dry Skull Group			Patient Group			Total		
	Total	Female	Male	Total	Female	Male	Total	Female	Male
**Mean (mm)**	10.32	9.97	10.59	10.00	9.65	10.28	10.09	9.74	10.37
**St. Dev.**	1.65408	1.41909	1.77253	1.67461	1.40372	1.81592	1.67402	1.41347	1.80725
**Min (mm)**	6.40	7.30	6.40	5.40	5.60	5.40	5.40	5.60	5.40
**Max (mm)**	15.40	13.80	15.40	17.00	13.60	17.00	17.00	13.80	17.00
**Total (no.)**	241	105	136	600	265	335	841	370	471

**Table 4:** The relationship between the SS pneumatization type and the dimensions of the ST.

	Dry Skull Group		Patient Group		Total	
	Pneumatization Type	r, *p*	Pneumatization Type	r, *p*	Pneumatization Type	r, *p*
**STH**	Choncal	0.14, *p* = 0.02 *	Choncal	0.08, *p* = 0.02 *	Choncal	0.08; *p* = 0.01 *
	Presellar	0.14, *p* = 0.02 *	Presellar	0.25, *p* = 0.001 *	Presellar	0.19, *p* = 0.001 *
	Sellar	0.13, *p* = 0.04 *	Sellar	0.15, *p* = 0.001 *	Sellar	0.14, *p* = 0.001 *
	Postsellar	−0.30, *p* = 0.001 *	Postsellar	−0.31, *p* = 0.001 *	Postsellar	−0.29, *p* = 0.001 *
**AP diam. ST**	Choncal	−0.03, *p* = 0.57	Choncal	−0.05, *p* = 0.19	Choncal	−0.03, *p* = 0.34
	Presellar	−0.01, *p* = 0.80	Presellar	−0.002, *p* = 0.80	Presellar	0.002, *p* = 0.96
	Sellar	−0.03, *p* = 0.63	Sellar	−0.05, *p* = 0.18	Sellar	−0.04, *p* = 0.15
	Postsellar	0.06, *p* = 0.35	Postsellar	0.06, *p* = 0.10	Postsellar	0.05, *p* = 0.09

* *p* < 0.05 exhibits a significant difference.

**Table 5:** Frequency of interclinoid osseous bridges by types, groups and sex.

		Dry Skull Group			Patient Group			Total		
		Total no. (%)	Female no. (%)	Male no. (%)	Total no. (%)	Female no. (%)	Male no. (%)	Total no. (%)	Female no. (%)	Male no. (%)
**Type I**	Absent	440 (88.0%)	192 (88.9%)	248 (87.3%)	510 (85.0%)	220 (83.0%)	290 (86.6%)	950 (86.4%)	412 (85.7%)	538 (86.9%)
	Left side	19 (3.8%)	7 (3.2%)	12 (4.2%)	26 (4.3%)	12 (4.5%)	14 (4.2%)	45 (4.10%)	19 (4.0%)	26 (4.2%)
	Right side	18 (3.6%)	8 (3.7%)	10 (3.4%)	39 (6.5%)	22 (8.3%)	17 (5.1%)	57 (5.2%)	30 (6.2%)	27 (4.4%)
	Bilateral	23 (4.6%)	9 (4.2%)	14 (4.9%)	25 (4.2%)	11 (4.2%)	14 (4.1%)	48 (4.4%)	20 (4.1%)	28 (4.5%)
	Total	500 (100%)	216 (100%)	284 (100%)	600 (100%)	265 (100%)	335 (100%)	1100 (100%)	481 (100%)	619 (100%)
**Type II**	Absent	467 (93.4%)	197 (91.1%)	270 (95.1%)	542 (90.3%)	239 (90.2%)	303 (90.4%)	1009 (91.7%)	436 (90.6%)	573 (92.6%)
	Left side	7 (1.4%)	4 (1.9%)	3 (1.1%)	18 (3.0%)	4 (1.5%)	14 (4.2%)	25 (2.3%)	8 (1.7%)	17 (2.7%)
	Right side	9 (1.8%)	3 (1.4%)	6 (2.1%)	14 (2.3%)	6 (2.3%)	8 (2.4%)	23 (2.1%)	9 (1.9%)	14 (2.3%)
	Bilateral	17 (3.4%)	12 (5.6%)	5 (1.7%)	26 (4.4%)	16 (6.0%)	10 (3.0%)	43 (3.9%)	28 (5.8%)	15 (2.4%)
	Total	500 (100%)	216 (100%)	284 (100%)	600 (100%)	265 (100%)	335 (100%)	1100 (100%)	481 (100%)	619 (100%)
**Type III**	Absent	490 (98.0%)	211 (97.7%)	279 (98.1%)	543 (90.5%)	238 (89.8%)	305 (91.0%)	1033 (93.9%)	449 (93.4%)	584 (94.4%)
	Left side	3 (0.6%)	2 (0.9%)	1 (0.4%)	22 (3.7%)	11 (4.2%)	11 (3.3%)	25 (2.3%)	13 (2.7%)	12 (1.9%)
	Right side	2 (0.4%)	1 (0.5%)	1 (0.4%)	23 (3.8%)	12 (4.5%)	11 (3.3%)	25 (2.3%)	13 (2.7%)	12 (1.9%)
	Bilateral	5 (1.0%)	2 (0.9%)	3 (1.1%)	12 (2.0%)	4 (1.5%)	8 (2.4%)	17 (1.5%)	6 (1.2%)	11 (1.8%)
	Total	500 (100%)	216 (100%)	284 (100%)	600 (100%)	265 (100%)	335 (100%)	1100 (100%)	481 (100%)	619 (100%)

## Data Availability

The data presented in this study are available upon request from the corresponding authors.
